# Autism comorbidities show elevated female‐to‐male odds ratios and are associated with the age of first autism diagnosis

**DOI:** 10.1111/acps.13345

**Published:** 2021-07-14

**Authors:** Eya‐Mist Rødgaard, Kristian Jensen, Kamilla Woznica Miskowiak, Laurent Mottron

**Affiliations:** ^1^ Department of Psychology University of Copenhagen København K Denmark; ^2^ Department of Psychiatry and Addictology Université de Montréal Montreal QC Canada; ^3^ Psychiatric Centre Copenhagen Rigshospitalet København Ø Denmark; ^4^ Centre de Recherche du CIUSSS‐NIM Hôpital Rivière‐des‐Prairies Montréal QC Canada

**Keywords:** autism, comorbidity, heterogeneity, sex bias

## Abstract

**Objective:**

To investigate the association between the comorbidity rates in autism and sex, birth year and the age at which autism was first diagnosed and compare the relative impact of each.

**Method:**

Using the Danish National Patient Registry, cumulative incidences up to the age of 16 for 11 comorbid conditions (psychosis, affective disorders, anxiety disorders, conduct disorder, eating disorders, obsessive‐compulsive disorder, attention‐deficit hyperactivity disorder, epilepsy, tic disorders, sleep disorders or intellectual disability) were calculated for individuals with autism (N = 16,126) and non‐autism individuals (N = 654,977). Individuals were further stratified based on the age at the first autism diagnoses and comorbid diagnoses up to the age of 16 were compared.

**Results:**

Most comorbidities were significantly associated with birth year and sex. Female/male odds ratios for 8 of 11 comorbid conditions were up to 67% higher than the corresponding odds ratios in the non‐autism population, including conditions that are generally more common in males than in females as well as conditions that are more common in females. All comorbidity rates were significantly associated with the age at the first autism diagnosis, which was a stronger predictor than sex and birth year for 8 conditions.

**Conclusions:**

Comorbidity rates for females exceed what would be expected based on the sex ratios among non‐autistic individuals, indicating that the association between autism and comorbidity is stronger in females. Comorbidity rates are also highly dependent on the age at the first autism diagnosis, which may contribute to autism heterogeneity in research and clinical practice.

**Significant outcomes**

- In addition to sex differences and temporal changes, comorbidity rates were strongly associated with the age at which autism was first diagnosed. Age of diagnosis may thus provide information on comorbidity risk in a clinical setting.
- Comorbidity rates in females exceeded what would be expected based on sex differences in prevalence in the non‐autism population, which suggests that comorbidity in females increases the likelihood of receiving an autism diagnosis more than is the case for males.
- Autism and comorbid conditions were often diagnosed closely after each other, suggesting that some individuals with autism may not be diagnosed until they develop a comorbid condition.

**Limitations**

- Some milder cases of depression and anxiety may have been treated by a family physician and thus may not be represented in the data used here.
- The present results are based on data from a single country and replication in other datasets is required to assess the generalizability of the findings.
- The study primarily focused on psychiatric conditions, and further research is needed to investigate whether similar patterns exist for other medical comorbidities.

## 1 INTRODUCTION

Autism is a neurodevelopmental condition where symptoms manifest in childhood. Historically, autism has been considered a rare condition, but the prevalence has increased markedly in recent decades.^1^, ^2^ The condition is diagnosed more frequently in males than in females, with recent studies indicating a sex ratio around 3:1.^3^ The term autism covers a highly heterogeneous group of individuals^4^ and recent findings suggest that individuals diagnosed with autism deviate less from the general population than in previous years,^5^, ^6^, ^7^ which could contribute to increasing heterogeneity within the autism population. Autism is reported to be associated with an increased risk of other conditions including affective conditions, anxiety, attention‐deficit hyperactivity disorder (ADHD) and psychotic conditions.^8^, ^9^ This likely contributes to the heterogeneous clinical presentation, the difficulties that an individual with autism is experiencing, as well as the responses to various interventions.^10^, ^11^

High heterogeneity has also been found among studies estimating the rates of comorbid conditions in the autism population. For example, a meta‐analysis of comorbidity rates found a 95% prediction interval for the comorbidity rate of anxiety to be 2%–48%.^8^ While differences in how a comorbid diagnosis is ascertained may explain some of this variability,^9^ it is likely that different groups within the current autism population are associated with different risks of a given comorbid condition. Gaining a better understanding of the variability in autism comorbidity might thus lead to a better understanding of autism heterogeneity in general.

Several factors may contribute to the variability in autism comorbidity rates (CR). First, CRs may vary over time, that is differ between birth year cohorts, as the prevalence of several psychiatric conditions have changed over time.^12^ Furthermore, it is likely that the temporal shift in how autism is diagnosed has resulted in changes in the composition of the autism population, which might also affect CRs. Second, there may be sex‐based differences in CRs since the prevalence of many psychiatric conditions are known to differ between males and females.^13^ The effects of time and sex on autism comorbidity have previously been investigated, but significant unexplained variability remains^8^, ^14^, ^15^ and additional sources of variation in comorbidity should thus be investigated. A factor that may further explain some of the observed heterogeneity in CRs is variation in the age at which autism is first diagnosed. Autism is generally viewed as a condition with an early childhood onset, but there is a significant number of individuals who are not diagnosed until a later age.^16^ As the age at the first autism diagnosis likely correlates with other aspects of variation in autism, it is also possible that differences in age of diagnosis can account for some variability in comorbidity rates. For example, different degrees of deviation or distinct biological subtypes of autism may each be associated with specific comorbidities,^17^ as well as with developmental patterns affecting how early an autism diagnosis would be given.

### 1.1 Aims of the study

Here, we performed a registry‐based study to investigate how autism comorbidity rates vary according to birth year, sex and the age at which autism was first diagnosed and compared the explanatory power of each of them. Additionally, we examined whether sex ratios in comorbidity rates deviate from the sex ratio of prevalence rates in the non‐autism population.

## 2 METHODS

### 2.1 Data

We analysed data from the Danish National Patient Registry (DNPR), which tracks all diagnoses given to in‐ and out‐patients in the Danish hospital system. All diagnoses are linked to specific individuals, which allows analysis of comorbidity patterns by combining diagnoses across distinct hospital contacts and facilitates the analysis of DNPR data based on personal data, such as sex and age. The DNPR contains diagnostic data from 1994 to 2018 based on the 10th version of the International Classification of Diseases (ICD‐10). Since our aim was to investigate the associations of birth year, sex and age of diagnosis with comorbidity rate, we had to limit our analyses to conditions with a relatively high prevalence in the autism population. The set of conditions that we investigated (Table 1) was preselected based on overrepresentation among individuals with autism in previous studies (eg ^7^, ^8^, ^9^, ^18^). We restricted the dataset to individuals born from January 1, 1993 to 31 December 31, 2002. Although diagnoses were only available from 1994, we included individuals born in 1993, as we did not expect that any of the diagnoses of interest in this study would be given in the first year of life. We restricted our analyses to diagnoses given before the age of 16 to allow comparison of the rates across birth year cohorts. Individuals born after 2002 were thus not included. The age of 16 was chosen as the cut‐off, as the incidence of several of the comorbid diagnoses of interest, for example eating disorders, was expected to increase significantly with the onset of adolescence.^19^

**TABLE 1:** Comorbidity by sex, birth year and age of autism diagnosis

Comorbidity	Males (N = 11,914)	Females (N = 4212	Sex LR (*p*‐value; corrected *p*‐value)	Birth year LR (*p*‐value; corrected *p*‐value)	Sex LR (*p*‐value; corrected *p*‐value)	Birth year LR (*p*‐value; corrected *p*‐value)	Age of diagnosis LR (*p*‐value; corrected *p*‐value)
Attention‐deficit hyperactive disorder (F90*)	4154 (35%)	1079 (26%)	143.5 (<2e‐16; <3e‐16)	168.0 (<2e‐16; <1e‐15)	98.1 (<2e‐16; <4e‐16)	154.2 (<2e‐16; <1e‐15)	252.4 (<2e‐16; <3e‐16)
Affective disorders (F30*, F31*, F32*, F33*, F34*)	925 (8%)	809 (19%)	451.4 (<2e‐16; <3e‐16)	7.6 (0.11; 0.13)	217.1 (<2e‐16; <4e‐16)	13.5 (0.009; 0.01)	523.9 (<2e‐16; <3e‐16)
Anxiety disorders (*F93, F40*, F41*)	1163 (10%)	817 (19%)	235.7 (<2e‐16; <3e‐16)	97.0 (<2e‐16; <1e‐15)	139.4 (<2e‐16; <4e‐16)	97.8 (<2e‐16; <1e‐15)	271.0 (<2e‐16; <3e‐16)
Conduct disorder (F91*)	561 (5%)	128 (3%)	12.8 (3e‐4; 4e‐4)	16.9 (0.002; 0.003)	10.2 (0.001; 0.001)	18.2 (0.001; 0.002)	23.6 (8e‐6; 1e‐5)
Eating disorders (F50*)	112 (1%)	291 (7%)	321.1 (<2e‐16; <3e‐16)	17.8 (0.001; 0.002)	286.2 (<2e‐16; <4e‐16)	10.3 (0.04; 0.05)	16.2 (3e‐4; 3e‐4)
Epilepsy (G40*)	733 (6%)	286 (7%)	3.3 (0.07; 0.09)	33.9 (7e‐7; 2e‐6)	13.9 (2e‐4; 2e‐4)	29.0 (8e‐6; 2e‐5)	233.0 (<2e‐16; <3e‐16)
Intellectual disability (F7*)	1981 (17%)	705 (17%)	0.3 (0.58; 0.58)	35.7 (3e‐7; 8e‐7)	18.9 (1e‐5; 1e‐5)	27.6 (1e‐5; 2e‐2)	956.7 (<2e‐16; <3e‐16)
Obsessive‐compulsive disorder (F42*)	545 (5%)	357 (8%)	115.8 (<2e‐16; <3e‐16)	3.4 (0.50; 0.55)	62.0 (4e‐15; 6e‐15)	4.4 (0.35; 0.39)	76.5 (<2e‐16; <3e‐16)
Psychotic disorders (F2*)	479 (4%)	321 (8%)	131.2 (<2e‐16; <3e‐16)	2.3 (0.69; 0.69)	57.6 (3e‐14; 5e‐14)	2.2 (0.69; 0.69)	96.3 (<2e‐16; <3e‐16)
Sleep disorders (F51*, G47*)	245 (2%)	96 (2%)	2.4 (0.12; 0.13)	30.0 (5e‐6; 9e‐6)	4.1 (0.04; 0.04)	29.5 (6e‐6; 2e‐5)	8.2 (0.02; 0.02)
Tic disorders (F95*)	1037 (9%)	166 (4%)	107.0 (<2e‐16; <3e‐16)	36.3 (2e‐7; 7e‐7)	92.1 (<2e‐16; <4e‐16)	33.7 (8e‐7; 3e‐6)	31.8 (1e‐7; 1e‐7)

Counts and percentages of comorbidity for males and females (first two columns) and comparison of comorbidity rate by sex and birth year (middle two columns) and sex, birth year and age of autism diagnosis (last three columns). The codes in parentheses indicate the ICD‐10 codes that were used when identifying comorbid conditions. Asterisks indicate that all sub‐diagnoses of the listed code were included. Likelihood ratios (LR) indicate how much the model fit improved by including a given factor in the model, where a high LR reflects a large improvement in model fit when including the given factor.

The data was extracted on August 24, 2020. Among the selected birth year cohorts (1993–2002), we identified 16,126 individuals who had received an autism diagnosis (F84.0, F84.1, F84.5, F84.8 or F84.9) before their 16th birthday. Total birth‐cohort sizes for the calculation of prevalence rates were retrieved from Statistics Denmark, which is a governmental institution tasked with keeping statistics of the Danish population. The total number of individuals in the included birth year cohorts was 671,103. Aggregated counts of individuals with and without autism diagnosed with each condition before age 16 are listed in Tables S1 and S2. Our analysis protocol was approved by the University of Copenhagen, Faculty of Social Sciences.

### 2.2 Data quality

Diagnoses in the DNPR are given by specialist medical doctors within the Danish hospital sector, whereas diagnoses given by, for example family physicians are not included. The data in the DNPR thus reflects that a qualified professional has deemed the patient to fulfil the diagnostic criteria at a given time. Several previous studies have reviewed detailed records and compared these to DNPR diagnoses. These studies have generally found good validity of diagnoses in the DNPR, for example for autism,^20^ obsessive‐compulsive disorder (OCD),^21^ ADHD,^22^ schizophrenia^23^ and depression,^24^ although some concerns have been raised about diagnoses given in psychiatric emergency departments^24^ (see Supporting Material for details).

### 2.3 Epidemiological calculations

Comorbidity rates were calculated as the cumulative incidence, from birth to the 16th birthday, among individuals who received an autism diagnosis in that same time span, regardless of which diagnosis was given first. Cumulative incidence rates were calculated separately for males and females and two‐year birth cohorts (from 1993–1994 to 2001–2002). The individuals diagnosed with autism before 16 years of age were further stratified based on the age at which they first received an autism diagnosis, that is during early childhood (0–5 years), mid‐childhood (6–10 years) or late childhood (11–15 years), and CRs were calculated for each group. Crucially, the CRs were calculated over the same 0–15 year age window for all three groups (Figure 3a).

For each case of a comorbidity, the date of the contact where autism was first diagnosed was compared to the date of the contact where the comorbid condition was first diagnosed. This information was used to calculate the fraction of comorbidity cases where autism and the comorbid condition were diagnosed less than 6 months apart.

### 2.4 Statistical analyses

Each comorbid condition was analysed separately. Binomial regression was used to statistically test whether CRs differed significantly according to the factors sex, birth year or age of the first autism diagnosis. Thus, a generalized linear model was fitted using the ‘glm’ function in R version 3.6.2. Analysis of deviation was performed through likelihood ratio tests using the ‘ANOVA’ function of the ‘car’ package version 3.0‐6.^25^ The association of each factor with CR was assessed by testing the main effect of each, while controlling for the two other factors. The relative impact of each factor in the CRs was quantified by the likelihood ratio. A high likelihood ratio indicates that the model fits the data better when the factor in question is included than when it is not. Differences in female/male ratios for each condition were assessed by testing the interaction effect between autism and sex. *P*‐values were corrected for multiple testing using the Benjamini‐Hochberg method.^26^

## 3 RESULTS

### 3.1 Birth cohorts and sex

**FIGURE 1:**
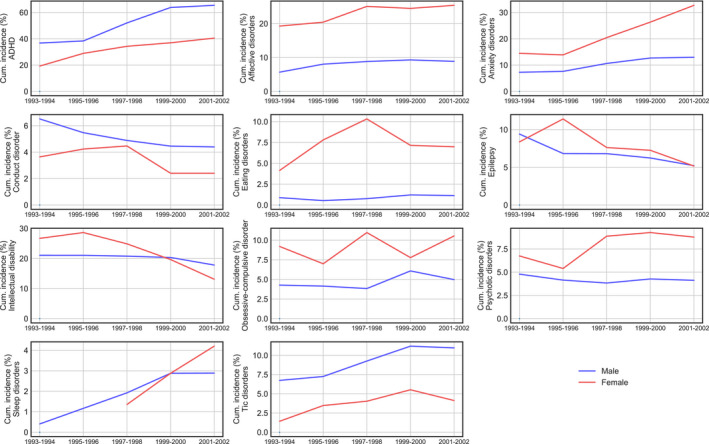
Comorbidity by birth year and sex. Cumulative incidence from birth to the 16th birthday per 100 individuals with autism, showing how comorbidity rates differ between two‐year birth cohorts for males and females

We first investigated the CR within five consecutive two‐year birth cohorts (Figure 1) and calculated whether they differed significantly between the sexes and between birth year cohorts (Table 1). Sex was statistically significant for 8 of 11 comorbid conditions, but not sleep disorders, intellectual disability (ID) or epilepsy. Similarly, CRs differed significantly between birth cohorts for 8 comorbid conditions.

### 3.2 Female/male odds ratios

**FIGURE 2:**
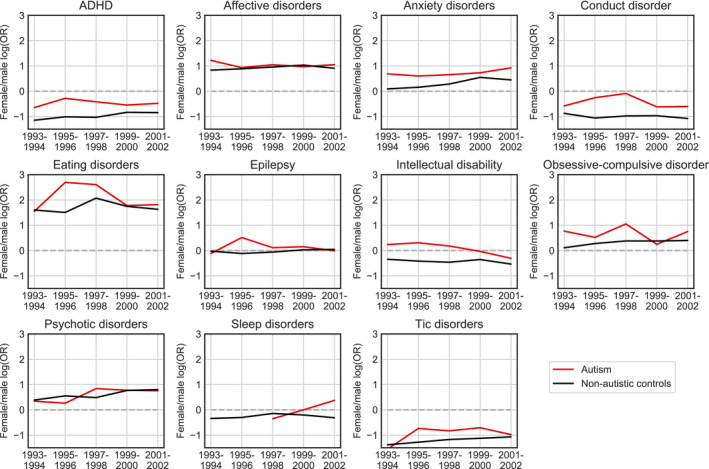
The female/male log odds ratios (ORs) for autism and non‐autism cohorts for each comorbid condition. Red lines indicate the female/male log OR for individuals with autism, while black lines indicate the female/male log OR for individuals without autism. The dashed lines indicate the point where males and females have equal risk

We calculated the female/male odds ratios (OR) for each condition for the autism cohorts and the corresponding non‐autism cohorts for each birth year (Figure 2). A binomial regression model was fitted to the data for each comorbid condition to assess whether the female/male OR’s for the autism and non‐autism cohorts significantly differed. The female/male OR was higher in the autism group than the non‐autism group for all conditions and the difference was significant for 8 of 11 conditions (Table 2), including conditions that are generally more common in males than in females as well as conditions that are more common in females. For example, among individuals with autism, the female/male OR for anxiety was approximately 2.2:1, whereas it was only approximately 1.4:1 for the non‐autism individuals. Autism is thus associated with a 58% increase in the female/male OR for anxiety (logOR = 0.46). Similarly, the female/male OR for ADHD was approximately 1:1.6 in autism, whereas it was 1:2.6 in the non‐autism population, revealing a 67% increase in the female/male OR in autism relative to the non‐autism population (logOR = 0.51).

**TABLE 2:** Differences in female/male odds ratios between the autism and non‐autism groups

Comorbidity	Sex ratio difference (Δlog odds ratio [95% CI])	*p*‐value (corrected *p*‐value)
Attention‐deficit hyperactive disorder	0.51 [0.42; 0.59]	<2e‐16 (<2e‐15)
Affective disorders	0.11 [−0.01; 0.23]	0.07 (0.08)
Anxiety disorders	0.46 [0.35; 0.57]	3e‐16 (<2e‐15)
Conduct disorder	0.53 [0.32; 0.74]	2e‐6 (4e‐6)
Eating disorders	0.34 [0.11; 0.58]	0.003 (0.004)
Epilepsy	0.14 [−0.01; 0.28]	0.07 (0.08)
Intellectual disability	0.43 [0.32; 0.54]	3e‐14 (1e‐13)
Obsessive‐compulsive disorder	0.33 [0.18; 0.49]	3e‐5 (6e‐5)
Psychotic disorders	0.06 [−0.10; 0.24]	0.44 (0.44)
Sleep disorders	0.63 [0.38; 0.88]	2e‐6 (4e‐6)
Tic disorders	0.33 [0.15; 0.52]	5e‐4 (7e‐4)

Positive sex ratio differences indicate that the female/male odds ratio (OR) is higher in the autism population than in the non‐autism population. The numbers in brackets indicate the 95% confidence intervals for the log OR differences, while the right column shows *p*‐values for the null hypothesis that Δlog(OR) = 0, as well as *p*‐values corrected for multiple testing.

### 3.3 Age of diagnosis

**FIGURE 3:**
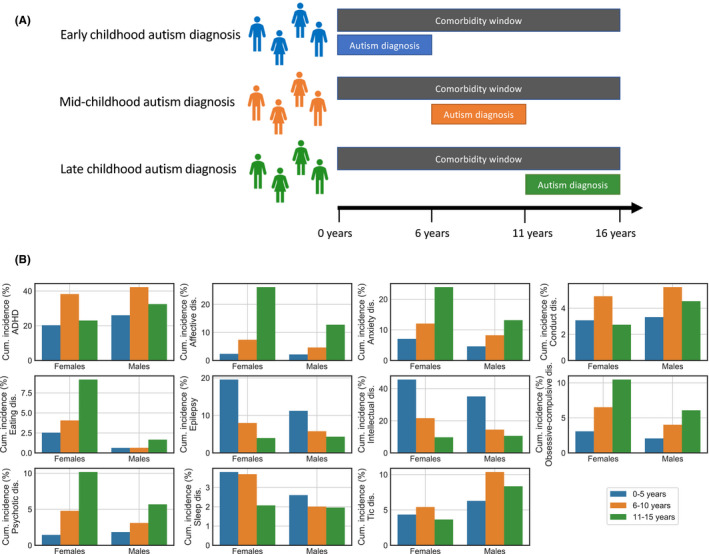
Comorbidity by age of diagnosis (A) Diagram of how the autism sample was stratified by age of first autism diagnosis. CRs were calculated over the age window of 0–15 years for all three groups. (B) Cumulative incidence from birth to 16th birthday per 100 individuals with autism, showing how CRs differ based on the age of first autism diagnosis for males and females, respectively. For simplicity, the data is aggregated across birth year cohorts. Figure S2 shows the data separated by birth year

In addition to sex and birth year, the autism cohorts were separated based on the age at which the first autism diagnosis was given, and CRs for the 0–15 years age period were calculated for each group (Figure 3). Differences in CR for each of the three factors were statistically tested (Table 1). The association between CR and age of autism diagnosis was significant for all comorbid conditions. The magnitude of the association between CR and age of autism diagnosis varied between comorbid conditions. For 8 out of 11 conditions, age of autism diagnosis had a larger impact on model goodness‐of‐fit (quantified by the likelihood ratio) than either sex or birth year. ID and epilepsy were diagnosed most frequently among the group diagnosed with autism in early childhood (0–5 years), whereas this group had lower rates of affective disorders and psychotic disorders. The group that was diagnosed with autism in mid‐childhood (6–10 years) had the highest frequencies of ADHD and tic disorder. The age group that was diagnosed with autism in late childhood (11–15 years) was diagnosed with psychotic disorders, affective disorders, OCD, eating disorder and anxiety more frequently than those diagnosed with autism in early or mid‐childhood. For each case of a comorbidity, we investigated whether the comorbid condition was first diagnosed within 6 months (before or after) of the first autism diagnosis. This was true for 56% of the cases of comorbidity (Figure S1).

## 4 DISCUSSION

We found birth year and sex to be significantly associated with differences in CRs for most of the investigated conditions. This is not surprising as the general prevalence of many psychiatric conditions is known to differ between sexes and to have changed over time.^12^, ^13^ However, both differences in birth year and sex contribute to the observed heterogeneity in autism comorbidity and are thus relevant to account for when mapping comorbid conditions associated with autism. The difference in CRs between birth year cohorts may reflect a change in the composition of the autism population in recent years as a result of increased autism prevalence and potential broadening of the autism diagnosis.^5^, ^6^ For example, we found a decrease in the CR of ID, as previously reported by Idring and coworkers,^7^ indicating that autism without intellectual impairment constitutes an increasing proportion of the autism population.

The observed sex‐based differences are largely consistent with previously reported differences in comorbidity patterns between males and females.^14^, ^15^ We generally found the female/male OR to have the same direction as in the non‐autism population (Figure 2), but the female/male OR was significantly higher among individuals with autism for 8 out of 11 conditions than in the non‐autism population, regardless of whether the condition was most common in males or females. Autism is thus associated with a disproportionately increased risk of comorbid diagnoses for females than males, further indicating that comorbidity studies should examine and account for sex‐based differences. The fact that a sex ratio disparity was observed across comorbid conditions indicates that the effect is caused by a common factor related to autism rather than distinct factors for each of the comorbid conditions. Such a factor may be either biological or diagnostic in nature. A similar pattern of females having a disproportionately higher risk for a range of comorbid conditions has previously been observed in individuals with ADHD,^27^ suggesting that a sex‐skewness in comorbidity could be a general feature of developmental conditions.

Furthermore, in all investigated conditions we found the age at which autism was first diagnosed to be a significant predictor for risk of comorbidity before the 16th birthday (Figure 3), and for 8 out of 11 conditions the age of autism diagnosis was a stronger predictor than sex and birth year. Comorbid conditions were tracked over the same 16‐year time span for all individuals regardless of age of first autism diagnosis (Figure 3a), and these findings are thus not simply caused by general differences in the age at which a condition occurs or is commonly diagnosable. For several conditions, there were striking differences in comorbidity rate depending on the age at the first autism diagnosis. For example, among those with a late autism diagnosis 26% of females and 13% of males were diagnosed with an affective disorder at a point during childhood (0–15 years). This was true for less than 3% of those with an early autism diagnosis, which was considerably closer to the non‐autism cohort (1%). In contrast, ID was diagnosed in around 40% of those with an early autism diagnosis, and only in around 10% of those with a late autism diagnosis. Although more research should address this issue for each condition individually, age of diagnosis appears to be a useful proxy for comorbidity risk and potentially other aspects of autism heterogeneity in research as well as in clinical practice.

### 4.1 Biological heterogeneity

The association between CR and age of autism diagnosis may stem from biological heterogeneity in the nature of autism, such as subgroups each associated with different comorbid conditions and differences in the onset of autism symptoms and sex ratios. Previous research has shown that epilepsy in autism is often found among individuals who also have ID^17^, ^28^ and that the co‐occurrence of autism and epilepsy is associated with higher levels of hyperactivity^29^ and symptom severity.^30^ There is also evidence that the proportion of females among individuals with autism with ID is higher than for autism as a whole,^3^, ^31^ consistent with our finding that the female/male OR for ID is higher among individuals with autism than in the general population. This could indicate the presence of an autism subgroup with a relatively large proportion of females that is associated with ID and epilepsy,^28^ for example syndromic conditions associated with rare deleterious genetic variants.^17^ If this subgroup were also associated with an early onset of noticeable autism symptoms, it could explain our finding that these conditions are both frequently found in those diagnosed with autism before the age of 6 years and relatively rarely in those diagnosed later in childhood.

The association between the age of autism diagnosis and CR for anxiety and affective disorders may be mediated by differences in intellectual ability. Individuals with autism with a higher IQ may be better at using coping strategies such as scripted conversation or socially appropriate body language which make their autism characteristics less visible, and make them appear more socially competent, resulting in a later diagnosis.^32^, ^33^ Furthermore, depression among individuals with autism is more often diagnosed in those with a higher IQ,^34^ possibly due to having better insight about their own social difficulties.^35^ Conversely, depression and anxiety in individuals with autism with a low IQ may often be missed because of difficulties in verbalizing their distress in a way that is recognized and diagnosed.^36^

Biological differences between those diagnosed with autism at different ages could also reflect a gradient of symptom presentations. For instance, individuals with large deviations in language development and/or abnormalities may be more likely to be diagnosed early, while those with smaller deviations could go undiagnosed longer. This is consistent with our finding that those diagnosed with autism in early childhood had higher rates of ID and epilepsy, both of which have been associated with more pronounced symptoms when they co‐occur with the autism condition.^17^ However, it is not clear why these individuals with a presumably high degree of symptoms would be less likely to later develop anxiety, psychosis or affective disorders than individuals who are diagnosed with autism later in childhood, as indicated by our results.

The finding that autism is associated with a disproportionately high increase in risk of comorbidity for females may be explained by biological differences in how autism affects males and females. Such biological sex differences have previously been proposed to explain the male preponderance in autism.^37^, ^38^, ^39^ One hypothesis posits that females have a higher threshold for autism‐associated etiological factors, that is that females have lower risk of autism compared to males with the same levels of autism‐causing factors such as genetic variants.^40^ Since autism‐associated genetic variants are often also associated with an increased risk of other psychiatric conditions,^41^, ^42^ this could explain why the females that do develop autism have higher risk of comorbidity, as they would generally have a higher load of the predisposing genetic variants.

### 4.2 Non‐biological heterogeneity

As this study examined trends in diagnostic records, the observed results may not necessarily be caused by biological effects but could reflect non‐biological patterns concerning the diagnostic process. In support of this interpretation, comorbidity most often occurred in the group diagnosed with autism in the same age range in which the given comorbid diagnosis would generally be given in the general population (Figure S3)^19^, ^43^, ^44^, ^45^ and 56% of comorbid diagnoses were given within six months of the same hospital contact as the first autism diagnosis.

Symptom overlap between different diagnostic categories may explain the tendency for autism to be diagnosed simultaneously with a comorbidity. For example, there are symptom similarities between autism and ADHD,^11^ which frequently co‐occur in those diagnosed in mid‐childhood and diagnostic instruments have been found to have only modest specificity in a clinical setting, with ADHD responsible for the largest disagreement between standardized diagnostic scores and clinical judgement.^46^ Similarly, several studies have found that scores of autistic traits are higher for individuals with depression or social anxiety than controls,^47^, ^48^ whereas the elevation of autism trait scores is smaller for individuals with depression in remission than for those not in remission.^49^ Some individuals who develop depression or anxiety in late childhood may thus mistakenly also be diagnosed with autism because certain symptoms resemble autism symptoms, such as a lack of emotional facial expression or social withdrawal. In contrast, when autism has been diagnosed in early childhood, diagnostic overshadowing might cause symptoms of, for example depression or anxiety that develop later to be attributed to autism instead of being diagnosed as comorbidity,^50^ which may contribute to the relatively low frequency of some comorbid conditions in this group.

A direct causal effect of autism diagnoses on the development of comorbid conditions may also contribute to the association between CRs and the age of autism diagnosis. For example, undiagnosed autism may lead to problems that could have been managed more effectively if an autism diagnosis had been given earlier.^51^ This could potentially result in vulnerability to depression and anxiety and explain why individuals diagnosed with autism in late childhood are diagnosed with depression and anxiety more frequently than those who received an autism diagnosis earlier.

The tendency for autism and comorbidities to be diagnosed closely after each other could be explained by the appearance of a comorbidity increasing the likelihood that a child be referred for psychiatric assessment, thus leading to the discovery of a previously unrecognized autism condition. This mechanism was also hypothesized by Joshi and coworkers,^52^ who found that youths who were diagnosed with autism after being referred to a paediatric psychiatric centre were often given several additional diagnoses, such as ADHD, depression and anxiety. Furthermore, Aggarwal & Angus^51^ found that when adolescents and young adults were diagnosed with autism, they had often initially been referred for assessment due to symptoms of mood disorders, anxiety or psychosis, which could support this hypothesis. This suggests that there might be a selection bias in the diagnosed autism population with a larger presence of comorbid conditions than would be the case if all true autism cases would have been identified. Such a selection bias may also contribute to the observed sex ratio disparity. A diagnostic sex bias has been suggested, requiring females to exhibit more symptoms to be recognized as having autism,^53^, ^54^ which would likely cause the comorbidity enrichment to be stronger among females than males, as females with autism without comorbidities would be even less likely to be diagnosed than their male counterparts.

### 4.3 Implications

The pronounced differences in childhood comorbidity rates between the groups diagnosed with autism at different ages could indicate biological differences between these groups. It is possible that the age of diagnosis also correlates with other aspects of autism symptomatology and an increased understanding of the interaction between age of diagnosis and symptom profile would be beneficial for research as well as for clinical practice. It is also relevant to investigate whether the difference in comorbidity rates of, for example affective disorders could partly reflect underdiagnosis within certain groups of the autism population, for example in those with low IQ and/or who may have difficulty verbalizing their symptoms.

The association between comorbidity and age of first autism diagnosis could also be driven by a tendency for autism and comorbid conditions to be diagnosed closely after each other, because the likelihood of being diagnosed with autism might increase by the presence of other conditions. This may be partly explained by autism cases that are not noticed, until a comorbid condition develops, possibly accelerated by problems that could have been managed if an autism diagnosis had been given earlier. These cases may benefit from improved identification of autism before serious problems occur. Alternatively, the results may be explained by ‘false positive’ autism diagnoses where conditions such as mood disorders, psychosis or eating disorders result in symptoms that are mistaken for autism or temporarily amplify existing sub‐clinical autism‐like traits. In such cases, it might be beneficial to defer autism diagnoses until after other conditions have been managed. More generally, it might be worthwhile to consider a stronger focus on differential diagnosis, where the level of certainty required for an autism diagnosis is heightened in the presence of other psychiatric conditions with symptoms that overlap with autism. The specificity of autism diagnoses may be improved by further research into how the validity of autism diagnoses is affected by the presence of other conditions. The explanations mentioned above are not mutually exclusive, and it is possible that each contributes partly to the patterns observed.

## CONFLICT OF INTEREST

KWM declares having received consultancy fees from Lundbeck A/S and Janssen‐Cilag A/S in the past three years. EMR, KJ and LM declare no conflicts of interest.

### PEER REVIEW

The peer review history for this article is available at https://publons.com/publon/10.1111/acps.13345.

## Supporting information

Supplementary Material

## Data Availability

The aggregated count data necessary to perform the analyses described in this manuscript are included in the supplementary material (Tables S1 and S2). The raw data in the Danish National Patient Registry cannot be shared. Researchers can apply for access to the raw data through the Danish Health Data Authority.
