# Supplementary material for: Autism comorbidities show elevated female‐to‐male odds ratios and are associated with the age of first autism diagnosis
Source: Acta Psychiatr Scand. 2021 Jul 14;144(5):475–86. doi: 10.1111/acps.13345 (PMC9292172; doi:10.1111/acps.13345)
Supplement: Supplementary file 1 — Supplementary Material [file ACPS-144-475-s001.docx]

# Supplementary Methods

## Calculation of comorbidity rates

Comorbidity rates (CRs) were calculated as the cumulative incidence rate, from birth to the 16^th^ birthday, of a given comorbid diagnosis among individuals who were given an autism diagnosis during the same time span. In other words, the comorbidity of a diagnosis was the fraction of individuals that were given an autism diagnosis before 16 years of age who also received the comorbid diagnosis before 16 years of age. Cumulative incidence rates were calculated separately for males and females and for each two-year birth cohort (from 1993-1994 to 2001-2002) by identifying the number of individuals in the cohort who were diagnosed with the given diagnosis any time before the age of 16 and dividing by the size of the cohort. As an example, the CR for depression among males born in 2000 was calculated as follows. The number of individuals in the cohort who were diagnosed with autism before their birthday in 2016 was identified. Then, the number of those individuals who were also diagnosed with depression before their birthday in 2016 was identified. The CR was then given as the latter number divided by the former.

## Validity of the diagnoses

Previous studies have found good validity in the DNPR, e.g., for autism (Lauritsen et al., 2010), OCD (Nissen et al., 2017), ADHD (Mohr-Jensen et al., 2016), schizophrenia (Vernal et al., 2018), and depression (Frederiksen et al., 2020). It has been reported that diagnoses from psychiatric emergency departments may be less valid (Frederiksen et al., 2020) based on examination of whether electronic journal records supported the given diagnoses. However, this observation is not necessarily a sign of low validity but could also partially reflect a lower level of detail in journals from psychiatric emergency departments. Furthermore, the DNPR data is likely biased towards diagnoses given to more severe cases of psychiatric conditions (since mild cases may be managed outside the hospital sector) and excluding diagnoses from psychiatric emergency departments could exacerbate this problem. Thus, we included diagnoses from psychiatric emergency departments. Across the investigated conditions, less than 2% of the included cases were only diagnosed in a psychiatric emergency department, and our overall findings were generally the same whether they were included or not (Tables S3-4).

## References

Frederiksen, L. H., Bilenberg, N., Andersen, L., Henriksen, N., Jørgensen, J., Steinhausen, H., & Wesselhoeft, R. (2020). The Validity of Child and Adolescent Depression Diagnoses in the Danish Psychiatric Central Research Register. *Acta Psychiatrica Scandinavica*, *November*, 1–11. https://doi.org/10.1111/acps.13258

Lauritsen, M. B., Jørgensen, M., Madsen, K. M., Lemcke, S., Toft, S., Grove, J., Schendel, D. E., & Thorsen, P. (2010). Validity of Childhood Autism in the Danish Psychiatric Central Register: Findings from a Cohort Sample Born 1990-1999. *Journal of Autism and Developmental Disorders*, *40*(2), 139–148. https://doi.org/10.1007/s10803-009-0818-0

Mohr-Jensen, C., Vinkel Koch, S., Briciet Lauritsen, M., & Steinhausen, H. C. (2016). The validity and reliability of the diagnosis of hyperkinetic disorders in the Danish Psychiatric Central Research Registry. *European Psychiatry*, *35*, 16–24. https://doi.org/10.1016/j.eurpsy.2016.01.2427

Nissen, J., Powell, S., Koch, S. V., Crowley, J. J., Matthiesen, M., Grice, D. E., Thomsen, P. H., & Parner, E. (2017). Diagnostic validity of early-onset obsessive-compulsive disorder in the Danish Psychiatric Central Register: Findings from a cohort sample. *BMJ Open*, *7*(9), 1–7. https://doi.org/10.1136/bmjopen-2017-017172

Vernal, D. L., Stenstrøm, A. D., Staal, N., Christensen, A. M. R., Ebbesen, C., Pagsberg, A. K., Correll, C. U., Nielsen, R. E., & Lauritsen, M. B. (2018). Validation study of the early onset schizophrenia diagnosis in the Danish Psychiatric Central Research Register. *European Child and Adolescent Psychiatry*, *27*(8), 965–975. https://doi.org/10.1007/s00787-017-1102-z

# Supplementary Figures

FigureS1. Overview of how often comorbid diagnosis were first given in a hospital contact within 6 months of the contact where the first autism diagnosis was given, or before or after this period.

FigureS2. Cumulative incidence for each comorbid condition, separated by birth year, sex, and age of first autism diagnosis. Blue lines represent individuals diagnosed with autism before the age of 6, orange lines individuals diagnosed with autism at ages 6-10, and green lines individuals diagnosed with autism at ages 11-15.

#

Figure S3: Age of first diagnosis in the general population. For each of the included comorbid conditions, the bar plot shows the distribution of age of first diagnosis among individuals with or without an autism diagnosis. The bar heights indicate the fractions of all individuals diagnosed before age 16 who were first diagnosed within each age interval. For example, out of all individuals who received an ADHD diagnosis before the 16^th^ birthday, around 10% received it before the 6^th^ birthday, while 45% received it at ages 6-10 years and another 45% at ages 11-15 years.

# Supplementary Tables

TableS1: Numbers of autistic and non-autistic individuals that were diagnosed with each comorbid condition before the 16^th^ birthday. Each row represents a part of the total cohort, separated based on birth year, sex, and the presence/absence of autism (diagnosed before the 16^th^ birthday). The column N shows the total number of individuals in each group.

| Birth year | Sex | Autism/ control | N | ADHD | Affective disorders | Anxiety disorders | Conduct disorder | Eating disorders | Epilepsy | Intellectual disability | Obsessive-compulsive disorder | Psychotic disorders | Sleep disorders | Tic disorders |
| --- | --- | --- | --- | --- | --- | --- | --- | --- | --- | --- | --- | --- | --- | --- |
| 1993-1994 | F | Autism | 427 | 69 | 69 | 54 | 15 | 17 | 33 | 90 | 36 | 27 | <5 | 6 |
| 1993-1994 | M | Autism | 1488 | 400 | 80 | 101 | 91 | 13 | 128 | 259 | 61 | 68 | 6 | 94 |
| 1995-1996 | F | Autism | 566 | 127 | 96 | 69 | 23 | 41 | 58 | 126 | 37 | 29 | <5 | 19 |
| 1995-1996 | M | Autism | 1908 | 529 | 142 | 135 | 99 | 10 | 122 | 332 | 76 | 76 | 22 | 129 |
| 1997-1998 | F | Autism | 748 | 191 | 150 | 127 | 32 | 70 | 53 | 149 | 74 | 61 | 10 | 29 |
| 1997-1998 | M | Autism | 2383 | 816 | 193 | 229 | 111 | 18 | 152 | 410 | 88 | 88 | 45 | 202 |
| 1999-2000 | F | Autism | 1108 | 299 | 218 | 231 | 26 | 74 | 75 | 182 | 80 | 94 | 31 | 58 |
| 1999-2000 | M | Autism | 2929 | 1141 | 249 | 330 | 125 | 35 | 172 | 495 | 168 | 120 | 82 | 295 |
| 2001-2002 | F | Autism | 1363 | 393 | 276 | 336 | 32 | 89 | 67 | 158 | 130 | 110 | 55 | 54 |
| 2001-2002 | M | Autism | 3206 | 1268 | 261 | 368 | 135 | 36 | 159 | 485 | 152 | 127 | 90 | 317 |
| 1993-1994 | F | Control | 66360 | 504 | 755 | 512 | 196 | 523 | 1035 | 382 | 254 | 206 | 160 | 110 |
| 1993-1994 | M | Control | 68760 | 1620 | 342 | 484 | 485 | 110 | 1100 | 558 | 236 | 145 | 233 | 452 |
| 1995-1996 | F | Control | 66138 | 740 | 779 | 562 | 162 | 659 | 1012 | 421 | 342 | 230 | 208 | 143 |
| 1995-1996 | M | Control | 68797 | 2076 | 336 | 500 | 484 | 153 | 1180 | 663 | 270 | 138 | 292 | 531 |
| 1997-1998 | F | Control | 64267 | 800 | 880 | 683 | 155 | 798 | 919 | 425 | 388 | 251 | 312 | 170 |
| 1997-1998 | M | Control | 66424 | 2268 | 352 | 533 | 424 | 105 | 1009 | 695 | 275 | 160 | 372 | 564 |
| 1999-2000 | F | Control | 63885 | 1088 | 1072 | 1035 | 133 | 816 | 952 | 445 | 472 | 321 | 378 | 211 |
| 1999-2000 | M | Control | 65382 | 2509 | 395 | 617 | 357 | 147 | 947 | 648 | 332 | 153 | 473 | 661 |
| 2001-2002 | F | Control | 61707 | 1104 | 884 | 1090 | 112 | 735 | 846 | 339 | 452 | 303 | 368 | 241 |
| 2001-2002 | M | Control | 63257 | 2573 | 369 | 719 | 336 | 149 | 830 | 592 | 312 | 140 | 514 | 714 |

TableS2: Numbers of autistic individuals diagnosed with each comorbid condition before the 16^th^ birthday. Each row represents a part of the total cohort, separated based on birth year, sex, the age at which autism was first diagnosed. The column N shows the total number of individuals in each group.

| Birth year | Age at first autism diagnosis (years) | Sex | N | ADHD | Affective disorders | Anxiety disorders | Conduct disorder | Eating disorders | Epilepsy | Intellectual disability | Obsessive-compulsive disorder | Psychotic disorders | Sleep disorders | Tic disorders |
| --- | --- | --- | --- | --- | --- | --- | --- | --- | --- | --- | --- | --- | --- | --- |
| 1993-1994 | 0-5 | F | 71 | 6 | <5 | <5 | <5 | <5 | 15 | 32 | <5 | <5 | <5 | <5 |
| 1993-1994 | 0-5 | M | 281 | 49 | <5 | 9 | 11 | <5 | 39 | 102 | 7 | 5 | <5 | 7 |
| 1993-1994 | 6-10 | F | 95 | 21 | 9 | 14 | 8 | <5 | 7 | 21 | 5 | <5 | <5 | <5 |
| 1993-1994 | 6-10 | M | 531 | 167 | 14 | 41 | 40 | <5 | 39 | 77 | 16 | 23 | 6 | 40 |
| 1993-1994 | 11-15 | F | 261 | 42 | 60 | 40 | 7 | 17 | 11 | 37 | 31 | 27 | <5 | 6 |
| 1993-1994 | 11-15 | M | 676 | 184 | 66 | 51 | 40 | 13 | 50 | 80 | 38 | 40 | <5 | 47 |
| 1995-1996 | 0-5 | F | 101 | 14 | <5 | 7 | <5 | 6 | 24 | 52 | <5 | <5 | <5 | <5 |
| 1995-1996 | 0-5 | M | 414 | 74 | 11 | 16 | 18 | <5 | 39 | 122 | 8 | 6 | 10 | 20 |
| 1995-1996 | 6-10 | F | 104 | 30 | 7 | 8 | 7 | <5 | 13 | 29 | <5 | <5 | <5 | 6 |
| 1995-1996 | 6-10 | M | 624 | 187 | 29 | 49 | 40 | <5 | 42 | 105 | 16 | 18 | 5 | 56 |
| 1995-1996 | 11-15 | F | 361 | 83 | 89 | 54 | 16 | 35 | 21 | 45 | 37 | 29 | <5 | 13 |
| 1995-1996 | 11-15 | M | 870 | 268 | 102 | 70 | 41 | 10 | 41 | 105 | 52 | 52 | 7 | 53 |
| 1997-1998 | 0-5 | F | 115 | 27 | <5 | 5 | 5 | <5 | 22 | 54 | <5 | <5 | <5 | 6 |
| 1997-1998 | 0-5 | M | 434 | 111 | 8 | 16 | 18 | <5 | 51 | 156 | 7 | 9 | 12 | 33 |
| 1997-1998 | 6-10 | F | 127 | 44 | 12 | 14 | 8 | <5 | 10 | 35 | 12 | 7 | <5 | 6 |
| 1997-1998 | 6-10 | M | 827 | 339 | 36 | 64 | 40 | <5 | 51 | 129 | 29 | 24 | 15 | 80 |
| 1997-1998 | 11-15 | F | 506 | 120 | 138 | 108 | 19 | 70 | 21 | 60 | 62 | 54 | 10 | 17 |
| 1997-1998 | 11-15 | M | 1122 | 366 | 149 | 149 | 53 | 18 | 50 | 125 | 52 | 55 | 18 | 89 |
| 1999-2000 | 0-5 | F | 133 | 25 | <5 | 15 | <5 | <5 | 31 | 65 | <5 | <5 | 7 | 6 |
| 1999-2000 | 0-5 | M | 521 | 159 | 14 | 37 | 15 | 7 | 69 | 198 | 17 | 11 | 17 | 35 |
| 1999-2000 | 6-10 | F | 230 | 99 | 15 | 22 | 5 | 11 | 16 | 48 | 16 | 12 | 7 | 18 |
| 1999-2000 | 6-10 | M | 1133 | 518 | 56 | 86 | 62 | 7 | 51 | 166 | 59 | 33 | 26 | 118 |
| 1999-2000 | 11-15 | F | 745 | 175 | 203 | 194 | 21 | 63 | 28 | 69 | 64 | 82 | 17 | 34 |
| 1999-2000 | 11-15 | M | 1275 | 464 | 179 | 207 | 48 | 21 | 52 | 131 | 92 | 76 | 39 | 142 |
| 2001-2002 | 0-5 | F | 132 | 40 | 5 | 10 | 5 | <5 | 16 | 49 | 9 | <5 | 7 | 8 |
| 2001-2002 | 0-5 | M | 577 | 187 | 12 | 25 | 12 | <5 | 52 | 205 | 7 | 10 | 18 | 45 |
| 2001-2002 | 6-10 | F | 257 | 117 | 17 | 40 | 12 | 13 | 19 | 43 | 17 | 12 | 18 | 12 |
| 2001-2002 | 6-10 | M | 1264 | 639 | 68 | 121 | 64 | 10 | 70 | 158 | 56 | 38 | 36 | 160 |
| 2001-2002 | 11-15 | F | 974 | 236 | 254 | 286 | 15 | 76 | 32 | 66 | 104 | 98 | 30 | 34 |
| 2001-2002 | 11-15 | M | 1365 | 442 | 181 | 222 | 59 | 26 | 37 | 122 | 89 | 79 | 36 | 112 |

TableS3: Results of statistical tests investigating whether comorbidity rate is significantly associated with sex and birth year (first two columns) and sex, birth year and age of autism diagnosis (last three columns), excluding diagnoses given in psychiatric emergency departments. LR = likelihood ratio. df = degrees of freedom. The codes in parentheses indicate the ICD-10 codes that were used when identifying comorbid conditions. Asterisks indicate that all subdiagnoses of the listed code were included.

|  | **Sex** | **Birth year** | **Sex** | **Birthyear** | **Age of diagnosis** |
| --- | --- | --- | --- | --- | --- |
| **Comorbidity** | **LR (df = 1) (p-value)** | **LR (df = 4) (p-value)** | **LR (df = 1) (p-value)** | **LR (df = 4) (p-value)** | **LR (df = 2) (p-value)** |
| Attention-deficit hyperactive disorder (F90*) | 142.8 (<2e-16) | 171.0 (<2e-16) | 97.5 (<2e-16) | 156.7 (<2e-16) | 251.2 (<2e-16) |
| Affective disorders (F30*, F31*, F32*, F33*, F34*) | 455.5 (<2e-16) | 4.86 (0.30) | 210.5 (<2e-16) | 9.3 (0.05) | 452.3 (<2e-16) |
| Anxiety disorders (*F93, F40*, F41*) | 222.1 (<2e-16) | 94.7 (<2e-16) | 131.8 (<2e-16) | 95.6 (<2e-16) | 255.1 (<2e-16) |
| Conduct disorder (F91*) | 13.6 (2e-4) | 19.5 (0.001) | 11.2 (8e-4) | 21.4 (2e-4) | 26.9 (1e-6) |
| Eating disorders (F50*) | 320.5 (<2e-16) | 20.0 (0.001) | 286.3 (<2e-16) | 11.4 (0.02) | 15.6 (4e-4) |
| Epilepsy (G40*) | 3.0 (0.09) | 34.9 (5e-7) | 13.1 (3e-4) | 30.1 (5e-6) | 233.7 (<2e-16) |
| Intellectual disability (F7*) | 0.18 (0.67) | 34.6 (6e-7) | 17.8 (2e-5) | 26.9 (2e-5) | 958.1 (<2e-16) |
| Obsessive-compulsive disorder (F42*) | 110.7 (<2e-16) | 3.3 (0.51) | 58.0 (3e-14) | 4.5 (0.34) | 77.5 (<2e-16) |
| Psychotic disorders (F2*) | 125.6 (<2e-16) | 2.2 (0.70) | 56.4 (6e-14) | 1.8 (0.77) | 86.1 (<2e-16) |
| Sleep disorders (F51*, G47*) | 2.2 (0.14) | 31.7 (2e-6) | 4.0 (0.05) | 31.1 (3e-6) | 9.1 (0.01) |
| Tic disorders (F95*) | 105.4 (<2e-16) | 35.2 (4e-7) | 90.2 (<2e-16) | 32.6 (1e-6) | 32.3 (1e-7) |

TableS4: Differences in female-to-male odds ratios between the autism and non-autism groups, excluding diagnoses given in psychiatric emergency departments. Positive sex ratio differences indicate that the female/male odds ratio (OR) is higher in the autism population than in the non-autism population. Missing data for sleep disorders and tic disorders is due to numbers of cases being too low to estimate the sex ratios. The numbers in brackets indicate the 95% confidence intervals for the log OR differences, while the numbers in parentheses are p-values for the null hypothesis that Δlog(OR) = 0.

| **Comorbidity** | **Sex ratio difference (Δlog odds ratio [95% CI])** | **LR (df = 1) (p-value)** |
| --- | --- | --- |
| Attention-deficit hyperactive disorder | 0.51 [0.43; 0.60] | 129.7 (<2e-16) |
| Affective disorders | 0.11 [-0.01; 0.23] | 3.37 (0.06) |
| Anxiety disorders | 0.45 [0.34; 0.56] | 64.25 (1e-15) |
| Conduct disorder | 0.51 [0.29; 0.73] | 19.24 (1e-5) |
| Eating disorders | 0.37 [0.13; 0.61] | 9.68 (0.002) |
| Epilepsy | 0.13 [-0.02; 0.28] | 2.88 (0.09) |
| Intellectual disability | 0.43 [0.32; 0.54] | 56.4 (6e-14) |
| Obsessive-compulsive disorder | 0.33 [0.17; 0.48] | 16.73 (4e-5) |
| Psychotic disorders | 0.08 [-0.09; 0.25] | 0.80 (0.37) |
| Sleep disorders | 0.63 [0.38; 0.88] | 22.36 (2e-06) |
| Tic disorders | 0.34 [0.16; 0.53] | 12.55 (3e-4) |
